# Characterization of Non-Nutritive Sweetener Intake in Rural Southwest Virginian Adults Living in a Health-Disparate Region

**DOI:** 10.3390/nu9070757

**Published:** 2017-07-14

**Authors:** Valisa E. Hedrick, Erin M. Passaro, Brenda M. Davy, Wen You, Jamie M. Zoellner

**Affiliations:** 1 Department of Human Nutrition, Foods, and Exercise, Virginia Tech, 295 West Campus Drive, Blacksburg, VA 24061, USA; perin92@vt.edu (E.M.P.); bdavy@vt.edu (B.M.D.); 2 Department of Agricultural and Applied Economics, Virginia Tech, 250 Drillfield Drive, Blacksburg, VA 24061, USA; wenyou@vt.edu; 3 Department of Public Health Sciences, Cancer Center without Walls, University of Virginia, 16 East Main St., Christiansburg, VA 24073, USA; jz9q@virginia.edu

**Keywords:** non-nutritive sweeteners, artificial sweeteners, dietary assessment, human nutrition, rural region

## Abstract

Few data assessing non-nutritive sweetener (NNS) intake are available, especially within rural, health-disparate populations, where obesity and related co-morbidities are prevalent. The objective of this study is to characterize NNS intake for this population and examine the variance in demographics, cardio-metabolic outcomes, and dietary intake between NNS consumers and non-consumers. A cross-sectional sample (*n* = 301) of Virginian adults from a randomized controlled trial (data collected from 2012 to 2014) targeting sugar-sweetened beverage (SSB) intake completed three 24-h dietary recalls, and demographics and cardio-metabolic measures were assessed. The frequency, types, and sources of NNS consumption were identified. Thirty-three percent of participants reported consuming NNS (*n* = 100). Sucralose was the largest contributor of mean daily NNS intake by weight (mg), followed by aspartame, acesulfame potassium, and saccharin. NNS in tabletop sweeteners, diet tea, and diet soda were the top contributors to absolute NNS intake. The most frequently consumed NNS sources were diet sodas, juice drinks, and tabletop sweeteners. Although mean body mass index (BMI) was greater for NNS consumers, they demonstrated significantly lower food, beverage, and SSB caloric intake and energy density, and higher overall dietary quality. It remains unclear whether NNS use plays a role in exacerbating weight gain. NNS consumers in this sample may have switched from drinking predominantly SSB to drinking some NNS beverages in an effort to cope with weight gain. Future studies should explore motivations for NNS use across a variety of weight and health categories.

## 1. Introduction

Replacing added sugars in the diet with non-nutritive sweeteners (NNS) to reduce caloric intake, body weight, and cardio-metabolic risk factors is a controversial topic [1]. Randomized controlled trials and observational studies have reported both positive and negative potential associations between NNS intake and numerous health outcomes, including weight loss [2,3,4,5,6,7,8], weight gain [9,10,11,12], physiological and intestinal changes [13,14,15,16,17,18,19,20,21,22,23,24,25], cardiovascular disease [10,12,26,27,28,29,30], and diabetes [12,31,32]. Yet, the interpretation of these associations is limited by inadequate characterization of NNS consumption. For example, dietary intake assessment tools [1] focus mainly on NNS beverage intake, specifically diet sodas [11,26,27,28,33], and typically do not specify type or beverage brand [9,10,29,32,33]. This approach limits determination of type of NNS consumed in products [1,34]. Because of these limitations, NNS intake is often underestimated [11,26,27,28,33]. Currently, there are six types of NNS approved for use in the U.S.: acesulfame-potassium, aspartame, saccharin, sucralose, neotame, and advantame. Two other sweeteners (stevia and luo han guo or monk fruit) have been determined as “generally recognized as safe” (GRAS). These sweeteners are present in many foods including diet beverages, desserts, baked goods, and chewing gum, and personal hygiene products (e.g., toothpaste and medications). To gain a better understanding of the potential health outcomes associated with NNS consumption, it is necessary to first understand NNS intake patterns, as well as the characteristics of NNS consumers.

Numerous studies have assessed NNS intake, along with demographic, cardio-metabolic, dietary, and lifestyle characteristics of NNS consumers. Consumers of NNS were typically older [9,28], Caucasian [10,12,28,29] women [9,11,28,32] with higher weight statuses [9,10,28,29,30,31,32], and had more chronic disease risks [29,30,31,32]. NNS consumers also tended to have higher levels of education [9,10,28] and socioeconomic status [9,10], and were more likely to engage in dieting behavior [9,31] than NNS non-consumers. Usually their diets were lower in overall calories, as well as lower in calories from carbohydrates, sugar and sugar-sweetened beverages (SSB), alcohol, and milk [9,28,31]. Sylvetsky et al., also corroborated these findings, in the first study to assess national consumption trends of NNS foods and beverages using National Health and Nutrition Examination Survey (NHANES) data [34]. These national trends showed that 28% of the U.S. population (33% of females and 25% of males) consumed NNS during 2007–2008 [34]. Of these consumers, 38% were ≥55 years old (29% were 39–54 and 15% were 18–34 years old), 36% were Caucasian (22% Black and 25% Hispanic), 36% were obese (30% overweight and 22% normal), and 38% were in the highest income tertile (28% middle and 22% low) [34]. Similarly, the *Food and Health Survey: Consumer Attitudes toward Food Safety, Nutrition & Health* [35] reported that among a nationally representative sample of 1057 U.S. adults (18–80 years old), 30% of consumers reported consumption of NNS in 2012 [35].

## 2. Purpose

The overall aim of this secondary analysis of Talking Health data [36,37] was to characterize NNS intake (frequency, types, and sources of sweetener) of adults living in rural southwest Virginia. A secondary aim was to explore differences between NNS consumers and NNS non-consumer for (1) demographic characteristics (sex, age, race, educational attainment, socioeconomic status, and health literacy); (2) anthropometrics (weight and body mass index (BMI)); (3) cardio-metabolic measures (glucose and lipid values); (4) dietary intake (energy, beverages, SSB, water, macronutrients, added sugar, alcohol, sodium, and energy density); and (5) dietary quality (Healthy Eating Index-2010 scores (HEI-2010)). 

An exploratory aim was to examine how Theory of Planned Behavior (TPB) constructs related to reducing SSB consumption differed between NNS consumers and non-consumers. The TPB explains and predicts behaviors using attitudes, subjective norms, and perceived behavioral control, and assumes that behavioral intention is the most important driving factor of a person’s behavior [38]. This exploratory aim seeks to examine whether the consumption of NNS impacts participants’ attitudes and behaviors related to SSB consumption.

## 3. Study Design and Subjects

This study utilizes baseline, cross-sectional data, collected from April 2012 to September 2014, from a large, community-based, randomized-controlled behavioral trial, known as Talking Health [36,37]. The primary purpose of Talking Health was to evaluate the effectiveness of a six-month behavioral intervention aimed at decreasing the consumption of SSB versus a matched contact comparison group aimed at increasing levels of physical activity. Participants randomized into the SSB reduction trial significantly reduced their SSB consumption over the six-month intervention by 950 kilojoules (227 kilocalories) per day, as compared to the physical activity group (decrease of 222 kilojoules (53 kilocalories)) [37]. 

Eligible participants were English-speaking adults (≥18 years old) consuming ≥200 kcal of SSB per day, with access to a telephone and no physical activity limitations [36]. SSB consumption was determined with the Beverage Intake Questionnaire (BEVQ-15), which is a quantitative food-frequency tool that evaluates the frequency and volume of consumption of different beverages over the previous month [39,40,41]. 

This study targeted adults residing in an eight-county rural region in southwest Virginia, with a goal to recruit participants of low socioeconomic and literacy levels. This region’s average (mean ± standard deviation) rurality status is 6.1 ± 2.5 out of 9 on the Rural-Urban Continuum Codes (where 1 = urban and 9 = very rural) [42]. Residents of this region are mostly Caucasian (95%), with a high school education or less (58%), and 18% live below the federal poverty line, according to the U.S. Census Bureau [43]. 

Trained Extension Program Assistants and/or community members assisted with recruitment of participants, however, recruitment methods were tailored to each county. Recruitment efforts at organizations serving low resource populations, like Head Start and the Departments of Public Health, were also prioritized. Other recruitment methods included flyers in community sites, newspaper postings, and recruitment postcards sent to addresses provided by a preexisting Cooperative Extension database and/or a mailing company that identified low socioeconomic communities [36]. The study was conducted according to the guidelines laid down in the Declaration of Helsinki and the Virginia Tech Institutional Review Board approved the study protocol. Participants provided written informed consent prior to enrollment. Participants received $25 after completing baseline data collection.

## 4. Methods

Baseline data collection included demographic characteristics (age, sex, ethnicity/race, income, education status, health literacy), anthropometrics (height, weight, BMI), cardio-metabolic measures (glucose and lipid values), TPB questions, and dietary intake (three 24-h dietary recalls) [36]. Height and weight were assessed in light clothing without shoes using a research-grade stadiometer and a calibrated digital Tanita scale (Model: 310GS). Health literacy status was assessed with the Newest Vital Sign; possible scores range from 0–6, with 0–3 indicating a low health literacy status and 4–6 indicating a high health literacy status [44]. Fasting blood samples were obtained via a routine finger stick using a One Touch Fine Point Lancet (Lifescan, Johnson & Johnson Company, Chesterbrook, Pennsylvania). A CardioChek system (Polymer Technology Systems, Inc., Indianapolis, India) was used to determine current blood glucose, cholesterol (total, low- and high-density lipoproteins), and triglyceride concentrations [45]; it is important to note that information regarding pre-existing health conditions was not collected.

To assess dietary intake, trained researchers, supervised by a PhD-level Registered Dietitian Nutritionist, conducted three non-consecutive 24-h dietary recalls within a two-week period, capturing two weekdays and one weekend day [46]. Interviewer-administered methods were used to collect dietary recalls, with one completed at baseline data collection in-person and the following two completed via unannounced telephone calls. Nutritional analysis software (Nutrition Data System for Research (NDS-R) 2011, University of Minnesota) was used to analyze the dietary recalls, and extract mean daily intake of dietary variables (energy, percent macronutrients, etc.) [36]. Through dietary recalls, dietary quality was calculated using HEI-2010 scores [47], which evaluates a person’s diet and their adherence to the 2010 Dietary Guidelines for Americans [48]. The HEI-2010 consists of twelve dietary factors including total fruit, whole fruit, total vegetables, dark-green vegetables and beans, whole grains, dairy, total protein foods, seafood and plant proteins, fatty acids, refined grains, sodium, and empty calories (solid fats, alcohol, and added sugars) [47]. Higher HEI-2010 scores (on a scale ranging from 0 to 100) indicate greater conformity to the 2010 Dietary Guidelines for Americans [48]. HEI-2010 scores can be further categorized into good (>80), needs improvement (51–80), or poor (<51) [47]. Energy density (kcal/g) was calculated using average daily energy intake and the average total weight of consumed dietary items derived from the dietary intake recalls.

NNS content in participants’ diets was calculated by NDS-R [49]. The average mg of NNS from foods, beverages, and NNS packets was extracted from the component/ingredient level of participants’ dietary intake and categorization of NNS consumers and non-consumers was determined [49]. In previous observational studies, classification of NNS consumers was determined solely from a participant’s beverage intake, specifically diet sodas [10,11,12,26,27,28,33]. However, this method does not adequately define the intake level to classify someone as a NNS consumer, as many foods also contribute to NNS intake. Thus, we utilized a novel method where a participant was considered a NNS consumer if they consumed an amount of NNS *equivalent* to NNS found in one fl oz of diet soda, from either foods or beverages on any given day. This intake level corresponds to three mg acesulfame potassium, seventeen mg aspartame, twelve mg saccharin, or six mg sucralose [49], which we believe is enough to be considered intentional intake rather than minute involuntary amounts. For example, if a participant only consumed five mg of sucralose from all foods and beverages in one day, that individual would not be considered a NNS consumer. Since these participants were already SSB consumers (by study design), NNS consumption of at least the *equivalent* of one fl oz of diet soda was considered intentional intake. Prevalent NNS types were identified, along with major dietary sources of NNS. Additionally, quantification of consumers’ average daily NNS intake by mg content was determined.

To assess TBP constructs related to SSB intake, a 16-item validated questionnaire (7-point scale) was administered which addressed attitudes, subjective norms, perceived behavioral control, and behavioral intentions towards SSB consumption [37,50,51,52] (Table 1).

### Data Analysis

Using SPSS (version 21.0 for Windows, 2012; IBM, Armonk, NY, USA), descriptive analyses were performed to determine NNS frequency, type, and food source (means ± standard deviations and frequencies). Differences in demographic characteristics, anthropometrics, cardio-metabolic measures, dietary quality, dietary intake, and TPB constructs for NNS consumers and non-consumers were assessed using independent *t*-tests (continuous variables) and χ^2^ tests (categorical variables). The significance level was set a priori at ≤0.05.

## 5. Results

### 5.1. Demographic Characteristics and Anthropometrics

Participants from Talking Health (*n* = 301) were primarily Caucasian (93%) females (81%) with a mean age of 41.8 ± 13.4 years and a mean BMI of 33.0 ± 9.1 kg/m^2^ (57% were obese (BMI ≥30 kg/m^2^)). Of the sample, 68% had greater than a high school degree. Mean annual income was $23,173 ± 17,145, but a large proportion of participants earned less than $14,999 (43%). Among participants, 33% (*n* = 100) reported consuming NNS (i.e., at a minimum, the equivalent of 1 fl oz of diet soda). Comparison of NNS consumers and NNS non-consumers (*n* = 201) revealed no significant differences between sex, age, race, education level, or household income. However, mean BMI was significantly higher for NNS consumers as compared to non-consumers (mean difference = 2.6 ± 1.2 kg/m^2^; *p* = 0.02) (Table 2).

### 5.2. Intake Frequency and Total Daily Intake of Non-Nutritive Sweeteners

Mean daily intake of all types of NNS was 869 ± 3553 mg. This value is equivalent to approximately 3.5 cans (12 fl oz) of diet soda. Sucralose contributed the most to mean daily NNS consumption (1034 ± 2788 mg), with 2.5 times more mg consumed than aspartame (414 ± 1815 mg), the second largest contributor to mean daily NNS intake. Acesulfame potassium (367 ± 2257 mg) contributed similarly to aspartame, while saccharin contributed very minimally (52 ± 52 mg).

### 5.3. Dietary Sources of Non-Nutritive Sweeteners

When examining the top contributors to absolute NNS intake in mg, among NNS consumers, it was found that tabletop NNS (i.e., NNS from packets or NNS added to foods or beverages after preparation) made up 37% of total NNS intake, followed by diet tea (34%), and diet soda (27%) (Table 3).

**Figure 1:**
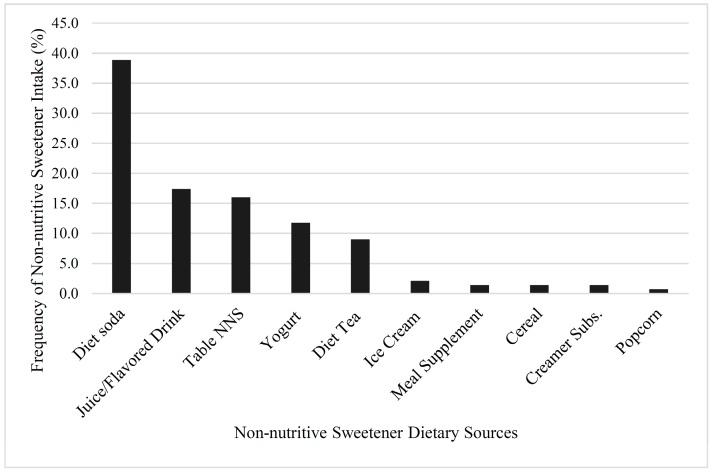
Dietary sources of non-nutritive sweetener (NNS) shown as percentage of total occurrences of NNS intake (*n* = 144) among NNS consumers (*n* = 100).

Among the 100 NNS consumers, there were 144 unique occurrences of NNS intake. For example, if an individual consumed two packets of sucralose, this was considered one occurrence. If the same individual also consumed a diet soda, then this was categorized as two occurrences of NNS intake. Thus, when examining the most frequently consumed dietary sources of NNS, it was found that diet soda was the most frequently consumed dietary source of NNS (39% of the 144 occurrences of intake), followed by juice and flavored drinks (17%), and tabletop NNS (16%) (Figure 1).

### 5.4. Cardio-Metabolic Measures

No significant differences were found in any cardio-metabolic measures between NNS consumers and non-consumers (Table 4).

### 5.5. Dietary Factors

Healthier dietary behaviors for NNS consumers versus non-consumers included significantly lower intake of total daily energy, total beverage energy, SSB (kcal and fl oz), total sugar (g), added sugar (% total kcal and g), alcohol (% total kcal), and energy density (kcal/g) (Table 5).

### 5.6. Dietary Quality

Dietary quality was assessed via HEI-2010 scores (Table 6). Overall, NNS consumers had significantly higher overall dietary quality than NNS non-consumers (46.7 ± 11.9 vs. 42.4 ± 12.6). Contributing to this higher score were significantly greater scores in total fruit, dark-green vegetables and beans, dairy, and empty calories (solid fats, alcohol, and added sugars) for NNS consumers. However, NNS consumers had significantly lower scores for refined grains and sodium. Other HEI subcomponents were not significantly different between NNS consumers and non-consumers.

### 5.7. Theory of Planned Behavior

When examining SSB TPB constructs, NNS consumers, as compared to non-consumers, had increased negative attitudes towards SSB consumption, higher social pressure (subjective norms) and perceived behavioral control for reducing SSB consumption, and greater intentions to decrease SSB consumption (Table 7).

## 6. Discussion

To characterize intake patterns in a rural population, frequency, type, and sources of NNS intake were investigated. Thirty-three percent of this sample consumed NNS. This proportion is fairly consistent with national NNS consumption rates [34,35], which report NNS consumers ranging from 28% to 30% of the population. However, it was expected that this sample’s NNS consumption would be lower due to reports of higher SSB intake among rural populations [53]. The high consumption rate found in this investigation could be a function of our NNS consumer definition (equivalent of 1 fl oz of diet soda) as compared to the traditional diet soda definition. Additionally, the higher intake could reflect a shift in beverage patterns among rural populations in order to cope with high obesity and chronic health disease risk [54,55,56,57]. When asked about NNS beverages, participants from rural southwest Virginia reported in a qualitative study (*n* = 54) that NNS beverages had mostly positive attributes, including taste and health outcomes, acknowledging that NNS beverages contained less calories and sugar [51]. Participants also explained that their doctor’s recommendations, along with a diagnosis of diabetes, would influence their consumption of NNS beverages [51]. Despite mostly positive feelings, negative attributes were mentioned, including concerns about cancer risk, as well as a dislike for the aftertaste of some NNS beverages. Overall, these qualitative findings support the idea that participants in this rural region believe the potential health benefits of NNS use outweigh the potential health consequences, and may have started to consume NNS in response to a weight or health problem. 

Sucralose was the most prevalent NNS type in this sample’s diet, followed by aspartame, acesulfame potassium, and saccharin. This corresponds well to NNS prevalence in food and beverage products, as sucralose is the most common, followed by very similar levels for acesulfame potassium and aspartame [58]. The most frequently consumed sources of NNS in this study were diet soda, juice and flavored drinks, and tabletop NNS, which correlated with national consumption trends that reported that diet soda was the most prevalent source of NNS, followed by reduced-calorie beverages (light fruit juices and lemonades) [34,35]. With previous study designs, participants only reported general details regarding their NNS use, such as the frequency and/or quantity of just diet soda intake [10,11,12,26,27,28,29,32,33]. This approach is problematic, as specific beverage types like juice drinks, mixes, teas, or other non-carbonated NNS beverages were not captured. Focusing on diet soda intake as the only NNS source underestimates NNS intake and limits the accuracy of NNS intake data, thereby decreasing the scope and understanding of the potential health benefits and/or problems.

These findings demonstrated that in terms of dietary intake, NNS consumers tended to have better overall dietary habits than non-consumers, particularly with regard to daily energy, SSB, total and added sugar intake, and energy density. However, NNS consumers had a significantly higher BMI than NNS non-consumers, which is consistent with findings from observational studies [9,10,28,29,30,31,32]. For example, Fowler et al., observed significantly greater gains in BMI over eight years for NNS consumers versus NNS non-consumers [9]. Conversely, several randomized controlled trials have found that the replacement of SSB with NNS consumption aids in weight loss over time [6,59].

In addition to consuming NNS, participants of the current study were also consuming at least 200 kcal of SSB daily. Consumption of SSB is associated with increased body weight [60] and cardiovascular disease risk [61,62]. Thus, another possible explanation for NNS consumers having a higher BMI status, despite better overall dietary quality, is that NNS consumers in this sample may have switched from drinking predominantly SSB to drinking some NNS beverages in an effort to cope with weight gain [9,63] or other health conditions, like diabetes or hypertension [31]. Corroborating this hypothesis is the increased perceived behavioral control and behavioral intentions that individuals reported in regard to reducing SSB consumption. Data derived from observational studies that report negative health outcomes with NNS consumption should be interpreted cautiously as the associations may be largely attributed to pre-existing conditions rather than NNS intake. Although we do not have information regarding participants’ pre-existing conditions, when looking at fasting glucose levels, 10% of participants were considered pre-diabetic or diabetic, which may have led to observed NNS consumption being higher than expected. Furthermore, it is not definitive whether or not NNS use plays a role in exacerbating weight gain and disease development, and it is important to note that this study was not designed to determine if the correlations are associative or causal in nature.

In terms of dietary quality, NNS consumers had a significantly better HEI-2010 total score, as well as significantly better scores for total fruit, dark green vegetables and beans, dairy, and empty calories. These results indicate NNS consumers’ diets in this sample adhered better to the 2010 Dietary Guidelines for Americans as compared to NNS non-consumers. Furthermore, NNS consumers had lower total energy intake and density, and consumed less energy from total beverages, SSB, total sugar, added sugar, and alcohol than non-consumers. Similarly, in an observational study by de Koning et al., NNS consumers had a better overall dietary quality than NNS non-consumers, consuming less red and processed meat and carbohydrate, and more protein, while also maintaining a lower total energy intake level [31]. Another observational study reported that NNS consumers were more likely to follow a healthier diet containing high amounts of fruit, whole grains, and milk [28]. However, HEI-2010 scores for refined grains and sodium were lower for NNS consumers. These values indicate that NNS consumers might be compensating for missed calories with foods that are highly processed and contain large amounts of sodium and refined grains. There is some speculation that individuals might justify consumption of a higher calorie food after consuming NNS [64], which could explain the lower HEI-2010 for refined grains and sodium. Another potential explanation for the higher intake of refined grains is the possibility that NNS created an enhanced preference for sweet taste due to the high sweetness levels [16].

This investigation is the first to characterize NNS intake (consumer characteristics and frequency, type, and source) in a large (*n* = 301) rural population. NNS intake patterns in rural populations are particularly relevant as these groups are at a higher risk for obesity and a variety of chronic health conditions [54,55,56,57]. This investigation is unique in that it explores the most commonly consumed types of NNS in both beverages and foods [1,34]. This differentiation between NNS type is important, as each type of NNS is composed of different compounds and is metabolized differently, which could affect health outcomes. Furthermore, employing our novel method to determine whether a participant was considered a NNS consumer by assessing NNS intake through both foods and beverages (i.e., consumption of at least the *equivalent* of one fl oz of diet soda) adds to the pre-existing NNS literature that currently has a heavy emphasis on NNS beverages, specifically diet sodas.

This investigation was not without limitations. Data from the Talking Health study were utilized, which aimed to evaluate the effectiveness of a six-month intervention to decrease SSB consumption. Thus, by design, these participants were high SSB consumers, which could affect their NNS intake, as well as the generalizability of our findings. However, rural populations are shown to consume high levels of SSB [53] and therefore, higher than average SSB consumption is expected in rural populations. Furthermore, we do not have information regarding pre-existing health conditions, which may confound the role of NNS on health. Misreporting and participant recall bias is a common limitation of self-reported dietary intake assessment methods [65]. Nevertheless, the study was supervised by a doctorate-level Registered Dietitian Nutritionist and interviewer-administered methods (including USDA’s automated multiple pass method) were employed to assess dietary intake, which helps combat misreporting in low health literate populations [66]. Additionally, nutritional databases have limited available NNS data. Thus, NNS intake may be underestimated in this sample due to the inability to analyze all dietary sources and types of NNS.

## 7. Conclusions

The characterization of NNS intake will facilitate the understanding of potential health outcomes associated with NNS consumption. To better understand the relationship between pre-existing health conditions and NNS use, future NNS characterization studies should explore motivations to use NNS across a variety of weight and health categories, as different motivations for NNS use are likely. Understanding motive factors for NNS use could help identify potential confounding variables, like high BMI in this study, allowing for a more complete comprehension of the role of NNS in the diet and its impact on health.

## Figures and Tables

**Table 1:** Theory of Planned Behavior (TPB) construct questions regarding sugar-sweetened beverage consumption.

TPB Construct	Questions (7-Point Scale)
	For you, drinking less than 1 cup of sugary drinks each day would be:
Attitudes	Enjoyable/unenjoyable Healthy/unhealthy Pleasant/unpleasant Wise/unwise Exciting/boring Beneficial/harmful
Subjective Norms	7. Most people who are important to you want you to drink less than 1 cup of sugary drinks each day: agree/disagree 8. For most people whose opinions you value, how would they feel about you drinking less than 1 cup of sugary drinks each day: approve/disapprove 9. Most people who are important to you will drink less than 1 cup of sugary drinks each day: true/untrue
Perceived Behavioral Control	10. You have complete personal control over limiting your sugary drinks to less than 1 cup each day, if you really wanted to: agree/disagree 11. Limiting your sugary drinks to less than 1 cup each day is mostly up to you if you wanted to: agree/disagree 12. Limiting your sugary drinks to less than 1 cup of sugary drinks each day if you wanted to do so would be: easy/difficult
Behavioral Intentions	13. You plan to limit your sugary drinks to less than 1 cup each day: agree/disagree 14. How many days per week do you intend to limit your sugary drinks to less than 1 cup (0–7) 15. How motivated are you to limit your sugary drinks to less than 1 cup each day: motivated/unmotivated 16. How determined are you to limit your sugary drinks to less than 1 cup each day: determined/undetermined

**Table 2:** Participant demographic characteristics by total sample and by non-nutritive sweetener (NNS) consumer status.

Characteristic	Total Samples (*n* = 301) *n* (%)	NNS Consumers (*n* = 100) *n* (%)	NNS Non-Consumers (*n* = 201) *n* (%)	Significance between NNS Consumers and Non-Consumers
Sex				χ^2^ = 0.67; *p* = 0.41
Male	56 (19)	16 (16)	40 (20)	
Female	245 (81)	84 (84)	161 (80)	
Mean age ± SD ^a^ (years)	41.8 ± 13.4	42.8 ± 13.5	41.4 ± 13.3	*t* = −0.87; *p* = 0.39
Race/Ethnicity				χ^2^ = 0.97; *p* = 0.81
White	280 (93)	92 (92)	188 (93.5)	
African American	13 (4)	5 (5)	8 (4)	
Other	8 (3)	3 (3)	5 (2.5)	
Mean Weight ± SD (kg)	90.5 ± 25.4	94.4 ± 28.6	88.5 ± 23.4	*t* = −1.92; *p* = 0.06
Mean Body Mass Index ± SD (kg/m^2^)	33.0 ± 9.1	34.7 ± 10.6	32.1 ± 8.2	*t* = −2.33; *p* = 0.02
BMI Category				χ^2^ = 0.09; *p* = 0.99
Underweight (≤18.4)	6 (2)	2 (2)	4 (2)	
Normal weight (18.5–24.9)	59 (19.5)	19 (19)	40 (20)	
Overweight (25–29.9)	65 (21.5)	21 (21)	44 (22)	
Obese (≥30)	171 (57)	58 (58)	113 (56)	
Education Level				χ^2^ = 0.08; *p* = 0.77
≤High school graduate	96 (32)	33 (33)	63 (31)	
≥Some college	205 (68)	67 (67)	138 (69)	
Mean Income ± SD ($)	23,173 ± 17,145	24,925 ± 18,022	22,301 ± 16,668	*t* = −1.25; *p* = 0.21
Mean Income ($)				χ^2^ = 1.83; *p* = 0.61
≤14,999	129 (43)	40 (40)	89 (44)	
15,000–34,999	96 (32)	30 (30)	66 (33)	
35,000–54,999	39 (13)	15 (15)	24 (12)	
≥55,000	37 (12)	15 (15)	22 (11)	
Mean NVS Score ± SD				
	4.0 ± 1.9	4.1 ± 1.9	3.9 ± 2.0	*t* = −0.84; *p* = 0.40

^a^ SD, Standard Deviation; ^b^ NVS, Newest Vital Sign (0–3 = low health literacy, 4–6 = high health literacy).

**Table 3:** Contribution of dietary sources to absolute non-nutritive sweetener (NNS) mg intake among NNS consumers (*n* = 100).

Dietary Sources of NNS	Sucralose mg (%)	Aspartame mg (%)	Acesulfame Potassium mg (%)	Saccharin mg (%)	Total NNS mg (%)
Tabletop Sweetener	36,942 (68)	200 (0.5)	0 (0)	348 (96)	37,490 (37)
Diet Tea	0 (0)	18,310 (61)	16,172 (92)	0 (0)	34,482 (34)
Diet soda	16,313 (30)	10,554 (35)	719 (4)	15 (4)	27,601 (27)
Juice and Flavored Drinks	546 (1)	385 (1)	429 (2.5)	0 (0)	1360 (1)
Yogurt	266 (0.5)	429 (1.5)	32 (0.2)	0 (0)	727 (0.7)
Meal Replacement Supplements	38 (0.07)	177 (0.5)	216 (1)	0 (0)	431 (0.4)
Ice Cream	0 (0)	104 (0.3)	2 (0.01)	0 (0)	106 (0.1)
Cereal	0 (0)	100 (0.3)	0 (0)	0 (0)	100 (0.1)
Coffee Cream Substitutes	15 (0.03)	0 (0)	14 (0.1)	0 (0)	29 (0.03)
Popcorn	22 (0.04)	0 (0)	0 (0)	0 (0)	22 (0.02)
Total (mg)	54,142	30,259	17,584	363	102,348

**Table 4:** Cardio-metabolic measures for non-nutritive sweetener (NNS) consumers versus NNS non-consumers.

Cardio-Metabolic Measure	NNS Consumers Mean ± SD ^a^ (*n* = 100)	NNS Non-Consumers Mean ± SD (*n* = 201)	Mean Difference ± SE ^b^
Glucose (mg/dL)	79.8 ± 26.3	78.0 ± 24.4	1.8 ± 3.1
Total Cholesterol (mg/dL)	163.0 ± 38.9	167.4 ± 36.4	4.4 ± 4.6
Low-density Lipoprotein (mg/dL)	94.4 ± 32.1	99.4 ± 31.7	5.0 ± 4.1
High-density Lipoprotein (mg/dL)	48.1 ± 15.5	45.1 ± 15.1	3.0 ± 1.9
Triglycerides (mg/dL)	122.2 ± 72.9	129.6 ± 77.6	7.4 ± 9.3

^a^ SD, Standard Deviation; ^b^ SE, Standard Error.

**Table 5:** Dietary factors for non-nutritive sweetener (NNS) consumers versus NNS non-consumers

Dietary Variables	NNS Consumers Mean ± SD ^a^ (*n* = 100)	NNS Non-Consumers Mean ± SD (*n* = 201)	Mean Difference ± SE ^b^
Total Energy (kcal)	1719 ± 671	1955 ± 990	235 ± 110 *
Total Beverage (kcal)	297 ± 217	476 ± 393	179 ± 42 ***
Total Beverage (fl oz)	63.4 ± 29.0	66.6 ± 36.9	3.3 ± 4.2
SSB (kcal)	238 ± 215	384 ± 361	146 ± 39 ***
SSB (fl oz)	21.8 ± 20.3	32.7 ± 30.0	11.0 ± 3.3 ***
Water (fl oz)	21.9 ± 21.2	24.6 ± 27.4	2.7 ± 3.1
Carbohydrate (% total kcal)	49.2 ± 8.8	51.4 ± 10.1	2.2 ± 1.2
Total Sugar (g)	97.3 ± 55.1	137.3 ± 92.9	40.0 ± 10.1 ***
Added Sugar (% total kcal)	16.7 ± 8.5	23.5 ± 12.4	6.8 ± 1.4 ***
Added Sugar (g)	74.1 ± 52.1	114.8 ± 87.6	40.7 ± 9.5 ***
Protein (% total kcal)	15.7 ± 3.7	14.5 ± 4.5	−1.3 ± 0.5 **
Fat (% total kcal)	34.8 ± 7.2	33.1 ± 7.4	−1.7 ± 0.9
Saturated Fat (% total kcal)	12.2 ± 3.3	11.4 ± 3.2	−0.7 ± 0.4
Alcohol (% total kcal)	0.2 ± 0.8	1.1 ± 4.2	0.9 ± 0.4 *
Sodium (mg)	3010 ± 1280	3089 ± 1477	79 ± 173
Energy Density (kcal/g)	0.7 ± 0.3	0.8 ± 0.3	0.1 ± 0.0 *

^a^ SD, Standard Deviation; ^b^ SE, Standard Error; * *p* ≤ 0.05; ** *p* ≤ 0.01; *** *p* ≤ 0.001.

**Table 6:** Dietary quality (Healthy Eating Index (HEI-2010)) for non-nutritive sweetener (NNS) consumers versus NNS non-consumers.

HEI-2010 Dietary Components (Maximum Score)	NNS Consumers Mean ± SD ^a^ (*n* = 100)	NNS Non-Consumers Mean ± SD (*n* = 201)	Mean Difference ± SE ^b^
Total Fruit (5)	1.5 ± 1.6	1.0 ± 1.5	−0.4 ± 0.2 *
Whole Fruit (5)	1.6 ± 1.9	1.1 ± 1.8	−0.5 ± 0.2 *
Total Vegetables (5)	2.8 ± 1.4	2.5 ± 1.5	−0.3 ± 0.2
Dark-green Vegetables and Beans (5)	1.5 ± 1.9	1.0 ± 1.6	−0.5 ± 0.2 *
Whole Grains (10)	2.6 ± 3.3	2.5 ± 3.2	−0.1 ± 0.4
Dairy (10)	5.4 ± 3.0	4.3 ± 2.8	−1.1 ± 0.4 ***
Total Protein Foods (5)	4.4 ± 1.0	4.2 ± 1.2	−0.1 ± 0.1
Seafood and Plants Proteins (5)	1.9 ± 2.1	1.7 ± 2.1	−0.2 ± 0.3
Fatty Acids (10)	4.0 ± 3.4	4.1 ± 3.3	0.1 ± 0.4
Refined Grains (10)	5.5 ± 3.3	6.6 ± 3.1	1.1 ± 0.4 **
Sodium (10)	3.1 ± 3.0	4.5 ± 3.1	1.4 ± 0.4 ***
Empty Calories (20)	12.4 ± 4.8	8.9 ± 5.6	−3.6 ± 0.7 ***
HEI Total Score (100)	46.7 ± 11.9	42.4 ± 12.6	−4.3 ± 1.5 **

^a^ SD, Standard Deviation; ^b^ SE, Standard Error; * *p* ≤ 0.05; ** *p* ≤ 0.01; *** *p* ≤ 0.001.

**Table 7:** Theory of Planned Behavior constructs regarding sugar-sweetened beverage intake for non-nutritive sweetener (NNS) consumers versus NNS non-consumers.

Theory of Planned Behavior Constructs Related to Sugar-Sweetened Beverage Consumption ^1^	NNS Consumers Mean ± SD ^a^ (*n* = 100)	NNS Non-Consumers Mean ± SD (*n* = 201)	Mean Difference ± SE ^b^
Attitudes	4.7 ± 1.0	4.5 ± 1.0	−0.3 ± 0.1 *
Subjective Norms	5.0 ± 1.4	4.6 ± 1.2	−0.4 ± 0.2 *
Perceived Behavioral Control	5.8 ± 1.1	5.1 ± 1.4	−0.6 ± 0.2 ***
Behavioral Intentions	5.3 ± 1.5	4.5 ± 1.6	−0.8 ± 0.2 ***

^1^ Constructs reported on a scale of 1–7; ^a^ SD, Standard Deviation; ^b^ SE, Standard Error; * *p* ≤ 0.05; *** *p* ≤ 0.001.

## Abbreviations

HEI-2010	Healthy Eating Index-2010
NHANES	National Health and Nutrition Examination Survey
NNS	non-nutritive sweeteners
SSB	Sugar-sweetened beverages
TPB	Theory of Planned Behavior
